# Supplementary figures and images for: A comparative study on immune responses to demineralized and decellularized bone substitute following intraperitoneal implantation in mouse model
Source: PLoS One. 2025 May 16;20(5):e0323666. doi: 10.1371/journal.pone.0323666 (PMC12084032; doi:10.1371/journal.pone.0323666)

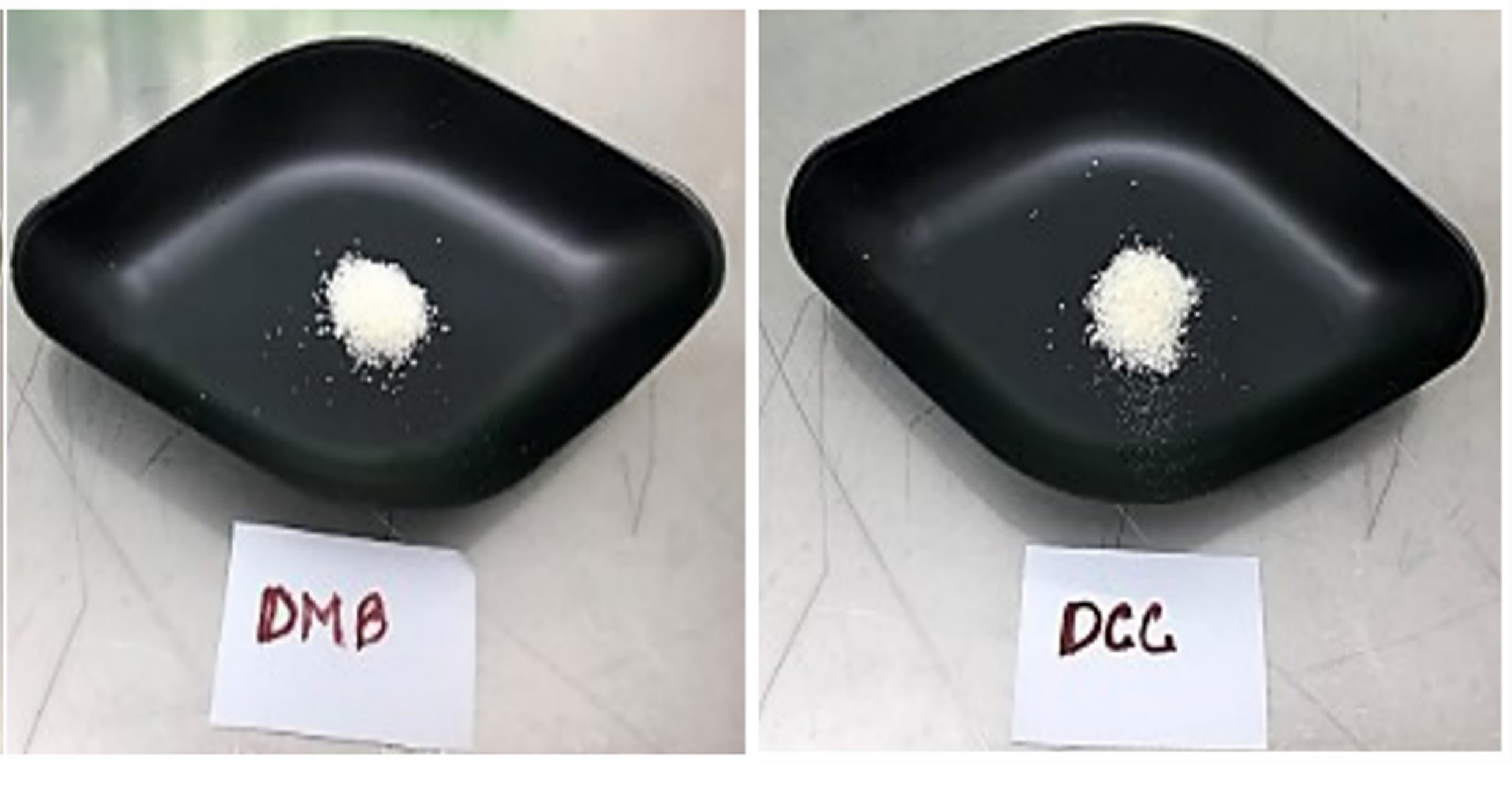

Supplement: S1 Fig — Granular form of bovine bone, DMB “Demineralized bone” and DCC “Decellularized bone” (TIF) [file pone.0323666.s001.tif]

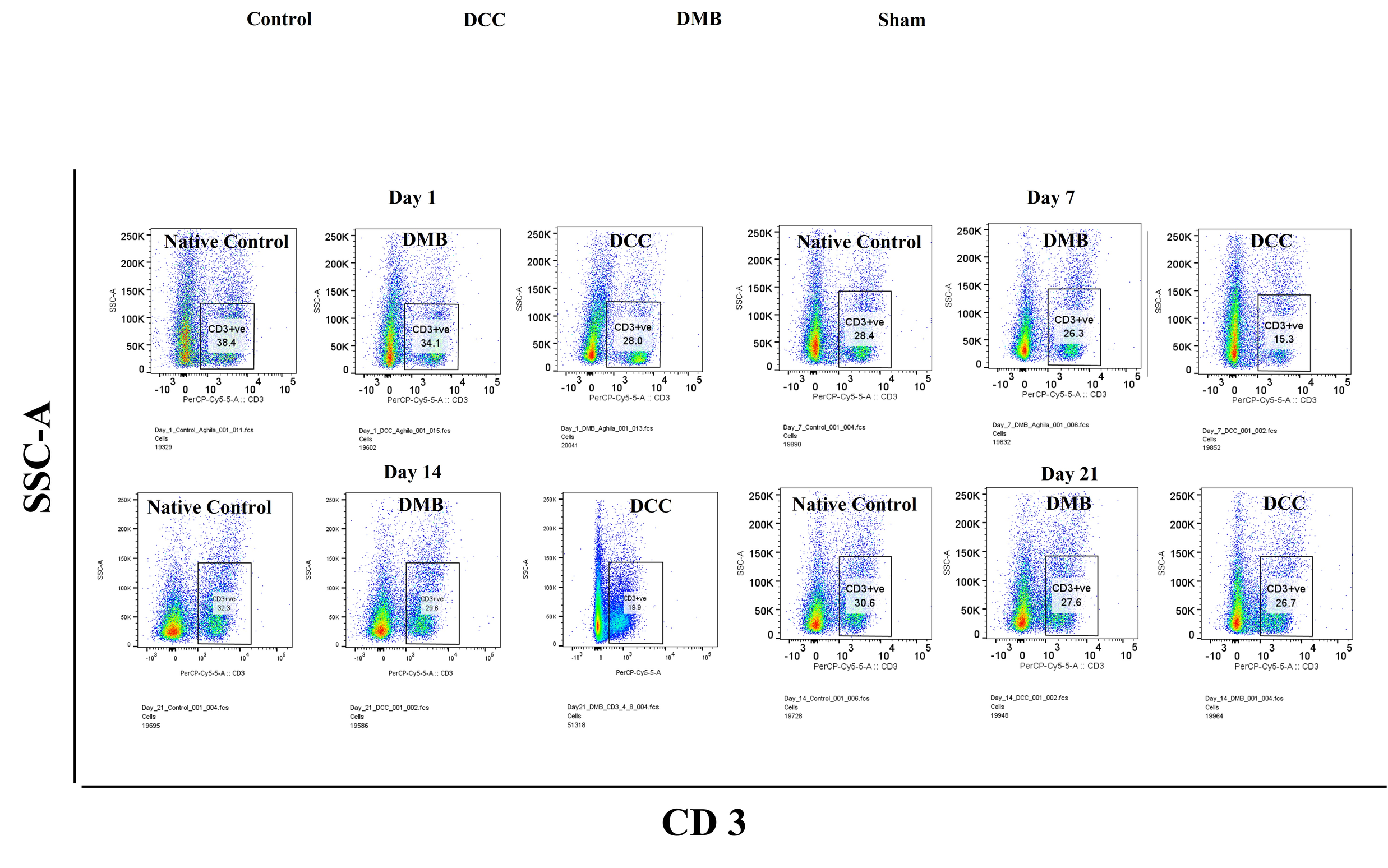

Supplement: S2 Fig — Flow cytometric analysis of CD3 + cells in spleens in native control bone, DMB, and DCC granules implanted mice at days 1, 7, 14, and 21. (TIF) [file pone.0323666.s002.tif]
