# Supplementary material for: A comparative study on immune responses to demineralized and decellularized bone substitute following intraperitoneal implantation in mouse model
Source: PLoS One. 2025 May 16;20(5):e0323666. doi: 10.1371/journal.pone.0323666 (PMC12084032; doi:10.1371/journal.pone.0323666)
Supplement: Attachment — Submitted filename: Response to Reviewers.docx [file pone.0323666.s004.docx]

| **Editor’s Comments** | **Responses** |
| --- | --- |
| 1. Please ensure that your manuscript meets PLOS ONE's style requirements, including those for file naming. The PLOS ONE style templates can be found at https://journals.plos.org/plosone/s/file?id=wjVg/PLOSOne_formatting_sample_main_body.pdf and  https://journals.plos.org/plosone/s/file?id=ba62/PLOSOne_formatting_sample_title_authors_affiliations.pdf | Manuscript meets PLOS ONE’s style requirements |
| 2. To comply with PLOS ONE submission requirements, in your Methods section, please provide additional information regarding the experiments involving animals and ensure you have included details on (1) methods of sacrifice, and (2) efforts to alleviate suffering. | Highlighted in page 6 & 7 |
| 3. We note that the grant information you provided in the ‘Funding Information’ and ‘Financial Disclosure’ sections do not match. When you resubmit, please ensure that you provide the correct grant numbers for the awards you received for your study in the ‘Funding Information’ section. | Added and corrected in “Funding information section”. |
| 4. Thank you for stating the following in the Acknowledgments Section of your manuscript:  “The authors expressed their gratitude to Ms. Razaz Omer of the Research Institute for Medical and Health Sciences for her contribution to Histological analyses. We would also like to acknowledge the funding resources from the University of Sharjah, UAE (grant no. 23001100169) awarded to Prof. Rani Samsudin.” | Thank you very much |
| 5. We note that you have provided funding information that is currently declared in your Funding Statement. However, funding information should not appear in the Acknowledgments section or other areas of your manuscript. We will only publish funding information present in the Funding Statement section of the online submission form.  Please remove any funding-related text from the manuscript and let us know how you would like to update your Funding Statement. Currently, your Funding Statement reads as follows:  “We would also like to acknowledge the funding resources from the University of Sharjah, UAE (grant no. 23001100169) awarded to Prof. Rani Samsudin.”   Please include your amended statements within your cover letter; we will change the online submission form on your behalf.   6. When completing the data availability statement of the submission form, you indicated that you will make your data available on acceptance. We strongly recommend all authors decide on a data sharing plan before acceptance, as the process can be lengthy and hold up publication timelines. Please note that, though access restrictions are acceptable now, your entire data will need to be made freely accessible if your manuscript is accepted for publication. This policy applies to all data except where public deposition would breach compliance with the protocol approved by your research ethics board. If you are unable to adhere to our open data policy, please kindly revise your statement to explain your reasoning and we will seek the editor's input on an exemption. Please be assured that, once you have provided your new statement, the assessment of your exemption will not hold up the peer review process. | As kindly advised, we have removed the funding information from the acknowledgement section of the manuscript.  We would like to retain the funding statement as *‘“We would also like to acknowledge the funding resources from the University of Sharjah, UAE (grant no. 23001100169) awarded to Prof. Rani Samsudin.”*  We have included the funding information in the cover letter and thank you for updating it in the online submission form.  We adhere to the open data policy and all data is contained within the manuscript.  We shall revise the statement in the online submission form. |
| 7. Please include captions for your Supporting Information files at the end of your manuscript, and update any in-text citations to match accordingly. Please see our Supporting Information guidelines for more information: http://journals.plos.org/plosone/s/supporting-information. | Added and highlighted in page 20 |
| **Reviewer 1** |  |
| 1. In this study Authors investigate the   immune responses following implantation of lyophilized demineralized (DMB) and lyophilized decellularized (DCC) bovine cancellous bone substitutes in a mouse peritoneal model. It assesses immunogenicity through hematological parameters, cytokine profiling, immune cell populations, and histological examination. The findings suggest that both DMB and DCC demonstrate favorable immunocompatibility, with DCC exhibiting superior immune tolerance.  This manuscript presents valuable findings on the immunogenicity of bone substitutes and is well-structured with rigorous experimental methods. However, it has some limitations, particularly regarding the small sample size, lack of long-term follow-up, and limited mechanistic insights. | Thank you very much for the valuable comments. We appreciate the recognition of the strengths of our study and the constructive feedback provided.  However, the small sample size was due to multiple time points and more than 1 group of comparison, so distribution of numbers of animals was among these different groups, and we adhere to the animal ethics protocol that emphasize using the minimum number of animals that may provide meaningful results.  Highlighted in pages 2,14 & 17.  For limited mechanistic insights, we have now added the gene expression studies for validating the differential expression of pro- and anti-inflammatory cytokines among the study groups that support the results we observed at the protein level (Fig 4 in the revised manuscript). |
| 2. The rationale for using an intraperitoneal implantation model rather than an orthotopic (bone defect) model is not well explained. Authors must provide a clearer justification for why this model was chosen over a direct bone implantation model, which may more accurately reflect clinical applications. | Highlighted and justified in page 17  In immunology testing for bone substitutes, the immune response can vary significantly depending on the site of implantation. Implanting bone substitutes in different tissues like the peritoneum or bone will lead to different immunological outcomes due to the distinct microenvironments of these sites. The peritoneal cavity is a site rich in immune cells, particularly macrophages, dendritic cells, and neutrophils, and it is part of the body’s primary defense system against foreign invaders. Implanting demineralized or decellularized bovine bone substitutes in the peritoneum leads to a rapid immune response, as shown in our study, since the peritoneal cavity is highly vascularized, and immune cells can quickly migrate to the site of implantation. In contrast, implantation of these bone substitutes in in-vivo bone defects may also initiate an immune response, but it often lead to a more delayed response, and less intense compared to implantation in soft tissues like the peritoneum. Thus bone substitutes implantation study in bone defects for shorter duration studies may demonstrate favorable ‘biocompatibility’ while the same material when implanted in the mice may show early immunologic response.  References (23,38,39) in page 20 and in references in pages 24 & 25 |
| 3. The study follows immune responses only up to 21 days, which may not capture the full spectrum of chronic immune reactions. Authors must consider extending the study duration to assess long-term immune responses and potential tissue integration. | Thank you very much for this suggestion. We shall consider continuing our study for longer duration in our future experiments to investigate the long-term immune responses.  The 21-day period is used in immunological studies to evaluate acute and early adaptive immune responses. This allows for the assessment of key inflammatory phases, including innate immune activation, early adaptive responses, and initial tissue remodeling. The primary objective of this study is to assess the initial immune reaction, which is crucial in determining early biocompatibility and potential adverse immune responses. While chronic immune responses are important, they are beyond the current study’s scope. However, future studies can build upon these findings to investigate long-term immune responses and tissue integration. |
| 4. The study lacks mechanistic exploration, particularly in detailing how DCC achieves better immunocompatibility at the molecular level. Authors must include gene expression analyses, to further elucidate pathways responsible for immune modulation. | We have explored and hypothesized the possible mechanisms of how DCC achieved better immunocompatibility at the molecular level through several previous experiments:  1. The residual nucleic acid in DCC is much lower than DMC thus promoting a much lower immunogenic response when exposed to human osteoblasts *invitro.*  2. DMB provoke a stronger immunologic response compared to DCC when exposed to human peripheral blood monocyte-derived macrophages (PBMM) suggesting decellularization process of tissues dampen down inflammatory reaction when exposed to PBMM.  **References:**   i) Al Qabbani A, Rani KGA, Syarif J, AlKawas S, Sheikh Abdul Hamid S, Samsudin AR, et al. (2023) Evaluation of decellularization process for developing osteogenic bovine cancellous bone scaffolds *in-vitro*. PLoS ONE 18(4): e0283922. <https://doi.org/10.1371/journal.pone.0283922> ii) Rani KGA, Al-Rawi AM, Al Qabbani A, AlKawas S, Mohammad MG, Samsudin AR (2024) Response of human peripheral blood monocyte derived macrophages (PBMM) to demineralized and decellularized bovine bone graft substitutes. PLoS ONE 19(4): e0300331. <https://doi.org/10.1371/journal.pone.0300331>   Additional gene expression study has been included in the result section pages 9 & 14 respectively & Fig 4 |
| 5. Authors must include comparative data or discuss how others bone graft materials, such as hydroxyapatite or allografts, perform relative to current clinical alternatives. | In this study, survival of all animals that remain active till the end of the implantation period suggest that both DMB and DCC excite a low inflammatory response that does not mount to lethal dose. When this immunological data was compared to other common biomaterials such as hydroxyapatite, a wide range of biocompatibility results were observed since the foreign body reaction to biomaterials also depend on the particle size, shape, topography and chemical composition and does not allow accurate comparisons when implanted in different body sites. While implantation studies of hydroxyapatite in bone defects tend to elicit a lower immune response because calcified structure is less prone to significant immune cell infiltration, similar material when implanted into soft tissue tend to provoke a much higher inflammatory response due to the more vascular and cellular soft tissue environment.  References:   1) Li Liu, Hao Chen, Xue Zhao et al. Advances in the application and research of biomaterials in promoting bone repair and regeneration through immune modulation. Materials Today Bio 30 (2025) <https://doi.org/10.1016/j.mtbio.2024>. 101410 2) James M. Anderson, Analiz Rodriguez and David T. Chang. Foreign body reaction to biomaterials. Seminars in Immunology 20 (2008) 86–100. doi:10.1016/j.smim.2007.11.004 3) **Ruhe, P. Q., Hedberg, E. L., Padron, N. T., Spauwen, P. H. M., Jansen, J. A., & Mikos, A. G. (2005).** "Biocompatibility and degradation of poly(DL-lactic-co-glycolic acid)/calcium phosphate cement composites. "Journal of Biomedical Materials Research Part A*,* 74A(4), 533-544. DOI: 10.1002/jbm.a.30318   As kindly suggested, we have included this information in the discussion of the revised manuscript (page 20). |
| **Reviewer 2** |  |
| 1. This study aimed to examine the immune responses of lyophilized demineralized (DMB) and lyophilized decellularized (DCC) bovine cancellous bone substitutes following implantation into the peritoneal cavity of BALB/c mice over a 21-day observation period. To assess the immunological responses and immunocompatibility of those bone substitutes, hematological assessments, spleen index measurements, immune cell responses, and cytokine profiling were performed, along with the histological examination of the spleen, liver, and kidney. | Thank you very much |
| 2. The manuscript is interesting and is well-organized, featuring robust experimental methods. Although this, there are several concerns that need to be addressed. The introduction and discussion are too long, they must be reduced. In particular, the discussion is redundant and must focus on results, novelty and limitations of the study. So, it must be completely revised. | Thank you very much for the comments, The introduction and discussion have been revised as advised and shortened accordingly. |
| 3. The authors should clarify why they chose the intraperitoneal implantation model. Being the manuscript merely descriptive, the authors must explore the molecular mechanism by which DCC bone substitutes promote superior immunocompatibility compared to DMB. The main text makes no reference to supplementary figure 2. Moreover, there are no legends of supplementary figures. | Highlighted and justified in page 17  In immunology testing for biomaterials, the immune response can vary significantly depending on the site of implantation. Implanting a biomaterial in different tissues like the peritoneum or bone will lead to different immunological outcomes due to the distinct microenvironments of these sites. In this study, the peritoneal model was chosen since this cavity is a site rich in immune cells, particularly macrophages, dendritic cells, and neutrophils, and it is part of the body’s primary defense system against foreign invaders. Implanting the bone substitutes in the peritoneum generally leads to a rapid immune response while testing them in immune-privileged sites such as in bone may generate minimal response and reflect a false impression of immune-compatibility.  Supplementary Fig 2 is referenced in page 13 of main text.  Legends of the supplementary figure have been added and highlighted at the end of the manuscript (page 20). |
